# Quasistatic Solutions versus Full-Wave Solutions of Single-Channel Circular RF Receive Coils on Phantoms of Varying Conductivities at 3 Tesla

**DOI:** 10.1155/2021/6638576

**Published:** 2021-04-29

**Authors:** Michael J. Beck, Dennis L. Parker, J. Rock Hadley

**Affiliations:** Department of Radiology and Imaging Sciences, Utah Center for Advanced Imaging Research, University of Utah, Salt Lake City 84132, USA

## Abstract

**Purpose.:**

Although full-wave simulations could be used to aid in RF coil design, the algorithms may be too slow for an iterative optimization algorithm. If quasistatic simulations are accurate within the design tolerance, then their use could reduce simulation time by orders of magnitude compared to full-wave simulations. This paper examines the accuracy of quasistatic and full-wave simulations at 3 Tesla.

**Methods.:**

Three sets of eight coils ranging from 3–10 cm (24 total) were used to measure SNR on three phantoms with conductivities of 0.3, 0.6, and 0.9 S/m. The phantom conductivities were chosen to represent those typically found in human tissues. The range of coil element sizes represents the sizes of coil elements seen in typical coil designs. SNR was determined using the magnetic and electric fields calculated by quasistatic and full-wave simulations. Each simulated SNR dataset was scaled to minimize the root mean squared error (RMSE) when compared against measured SNR data. In addition, the noise values calculated by each simulation were compared against benchtop measured noise values.

**Results.:**

The RMSE was 0.285 and 0.087 for the quasistatic and full-wave simulations, respectively. The maximum and minimum quotient values, when taking the ratio of simulated to measured SNR values, were 1.69 and 0.20 for the quasistatic simulations and 1.29 and 0.75 for the full-wave simulations, respectively. The ratio ranges, for the calculated quasistatic and full-wave total noise values compared to benchtop measured noise values, were 0.83–1.06 and 0.27–3.02, respectively.

**Conclusions.:**

Full-wave simulations were on average 3x more accurate than the quasistatic simulations. Full-wave simulations were more accurate in characterizing the wave effects within the sample, though they were not able to fully account for the skin effect when calculating coil noise.

## 1. Introduction

RF coil design for magnetic resonance imaging (MRI) applications is often an empirical process where the coil designer iteratively modifies and tests an RF-phased array coil design on the scanner until a satisfactory signal-to-noise ratio (SNR) is achieved in the region of interest (ROI) [1]. The process can be improved if, before coil construction and testing on the scanner, simulations are performed to allow the coil designer to estimate SNR in the ROI, so fewer or no iterations are required during the physical construction and testing phase of the design process [2]. Repeated testing of physically constructed coils can be very expensive, so designing phased array coils with the aid of simulation design tools could significantly reduce cost by shortening the testing phase on the scanner. [1, 2].

Significant progress has been made towards simulating phased array coil SNR [3–6]. MRI signal reception theory has been well established [7]; however, this theory did not account for phase changes in the sample. Hoult was the first to publish MRI signal reception theory that demonstrated how to derive MRI signal calculations with phase changes accounted for in the sample [3]. The signal reception equations derived by Hoult assumed one isochromat per voxel, and Jochimson et al. reduced the simulation time and error, due to one isochromat per voxel, when calculating the free induction decay of the receive signal. This was achieved by calculating the isochromat phase gradient between voxels in all directions [6]. Noise in a single coil element is characterized by Johnson noise. Roemer et al. were the first to derive a method for calculating the SNR when reconstructing a composite image from a phased array with multiple coil elements [8].

Magnetic and electric fields may be described in either the static, quasistatic, or high-frequency regime [9]. Maxwell’s equations describe the most intricate electromagnetic wave phenomena involving short time scales or high frequencies. Numerical methods that provide full-wave solutions of Maxwell’s equations include finite difference time domain (FDTD), finite-integral technique (FIT), finite-element method (FEM), and method of moments (MOM) [10–20]. Because quasistatic solutions can be calculated at rates that are orders of magnitude faster than the rates of full-wave numerical solutions, the use of numerical methods to solve for magnetic and electric fields may be unduly laborious if the fields can be obtained with sufficient accuracy using quasistatic methods. In addition, satisfactory quasistatic solutions can be found within a reasonable time frame using an optimization algorithm [21–23].

Quasistatic solutions are approximate solutions to Maxwell’s equations, but under the right conditions, the quasistatic solutions can approach exact solutions to Maxwell’s equations. The quasistatic equations are derived by assuming that electromagnetic waves propagate instantaneously with no retarded time. In other words, they are derived assuming the speed of light is infinite. Quasistatic solutions assume that no radiation or wave effects are present and that “the system is small compared to the electromagnetic wavelength associated with the dominant time scale [9].” There are three major quasistatic models for electromagnetics. They are the EQS (electroquasistatic), MQS (magnetoquasistatic), and the Darwin model [9,24]. The EQS model includes capacitive but not inductive effects, the MQS model includes inductive but not capacitive effects, and the Darwin model includes both inductive and capacitive effects. Because MRI RF systems are primarily designed to produce magnetic fields, only the magnetoquasistatic case will be considered in this paper [8].

If quasistatic solutions could be used at 3 Tesla to design phased array coils, it would greatly simplify and speed up the design process. For this to be the case, quasistatic solutions would need to be accurate within the design tolerance. Full-wave solutions are the gold standard for high-accuracy electromagnetic simulations, so it would be ideal to determine the accuracy of quasistatic solutions relative to full-wave solutions at 3 Tesla. To the author’s knowledge, no papers have been published that thoroughly compare quasistatic solutions to those found numerically at 3 Tesla.

The goal of this paper is to determine the accuracy with which a quasistatic solution can be used to predict the SNR distribution in the RF coil design process at 3 Tesla. The magnetic and electric field solutions obtained from quasistatic and full-wave simulations are used to determine MRI receive signal, noise, and resulting SNR. SNR is calculated for three sets of eight single loop coils on three cylindrical phantoms, each phantom varying in conductivity. These simulated SNR distributions are compared against the measured SNR distributions. Simulated noise values from each regime are compared against benchtop measured noise values. Lastly, four phantoms of varying sizes were simulated to show how phantom size affects each simulation method.

## 2. Methods

SNR data, determined through quasistatic and full-wave field solutions, were compared against SNR data measured on the scanner. Magnetic and electric fields were calculated using the MQS equations and numerically calculated using the FIT method. These simulations were performed with three sets of eight coils; the diameter of each of the eight coils varied in size to cover the range of coil-to-sample load ratios of coils typically found in phased array designs at 3 Tesla. These coils were simulated on three phantoms with varying conductivities that represent those conductivities typically found in human tissue. For comparison, three sets of eight coils were constructed, and actual measurements were made on phantoms with the same three conductivities. Simulated and benchtop measured noise values were compared for each coil element. Lastly, four phantoms of varying size were simulated to show how phantom size affects each simulation method; these were not compared against experiments.

### 2.1. Common Setup.

The commonalities between the MQS, full-wave, and measurement setups were as follows: each setup consisted of 3 sets of eight coil elements (24 in total). Each set of coil elements was comprised of a 3, 4, 5, 6, 7, 8, 9, and 10 cm diameter circular coil element. Each coil element had the following number of tune capacitors that were equally spaced: 1 (3 and 4 cm diameter coil elements only), 2 (5 and 6 cm diameter coil elements only), 3 (7 and 8 cm diameter coil elements only), and 4 (9 and 10 cm diameter coil elements only). Each coil element set for the full-wave and measurement setup were tuned and matched to either the 0.3, 0.6, or 0.9 S/m phantom, and each coil element was constructed using 16 AWG copper wire. The MQS simulations could not meet this requirement due to the required assumption that coil elements be line segments with no tuning capacitors, meaning coil elements simulated using the MQS equations were not tuned or matched.

**Figure 1:**
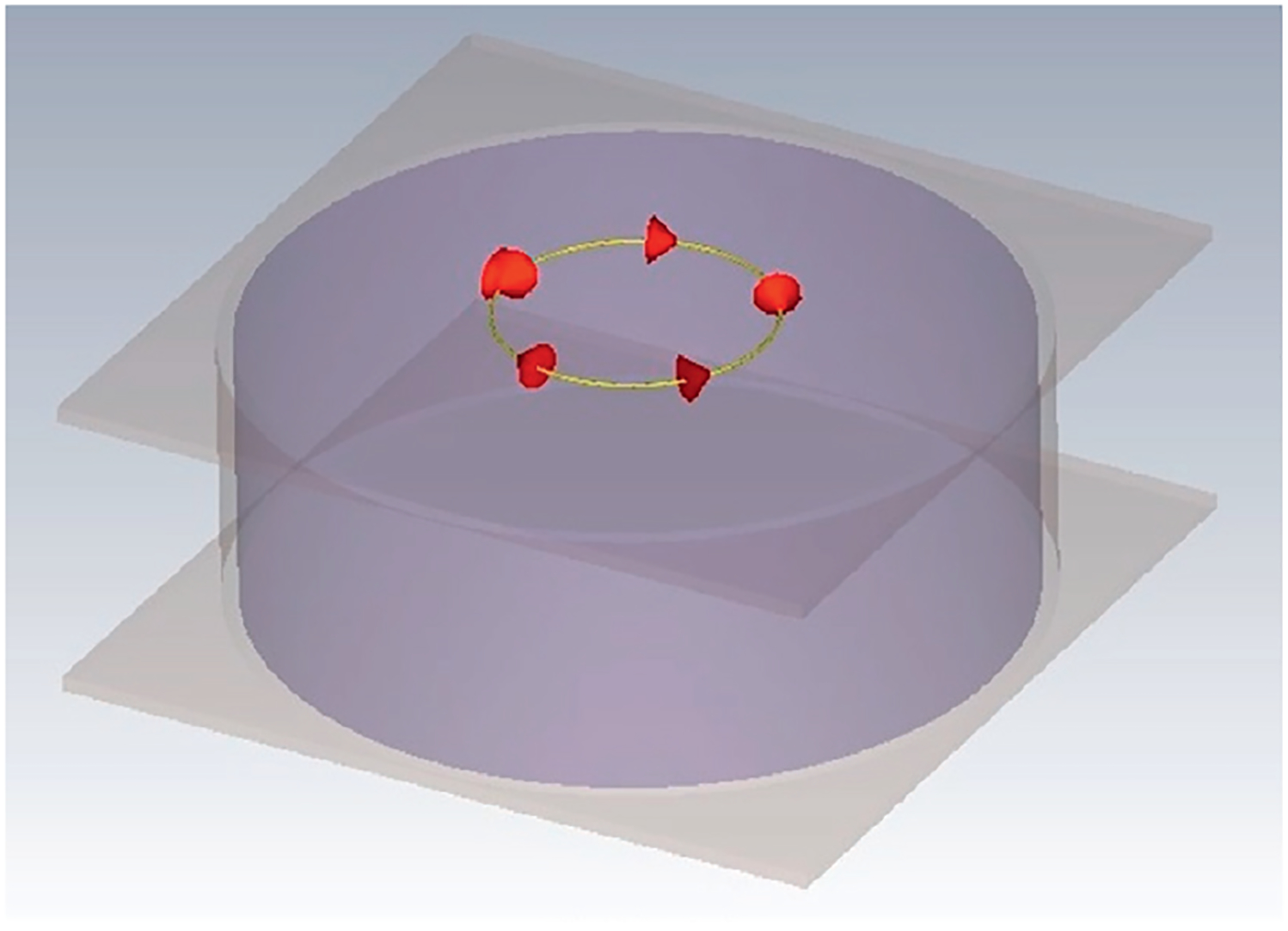
Three geometrically identical phantoms with conductivities of 0.3, 0.6, and 0.9 S/m were used. The phantom shown has a diameter/height of 27.8/10.1 cm with a 6 cm coil element. The ports for the match capacitor, tune capacitors, inductor, and excitation are also shown.

The same phantom setup was used for all simulations and measurements. Each setup used three cylindrical phantoms with coils centered on the phantom, as seen in Figure 1. The phantoms had conductivities of 0.3, 0.6, and 0.9 S/m at 20°C and a permittivity of 78. The solution height and radius were 10.1 and 13.9 cm, respectively. These measurements do not include the lossy polycarbonate material that contained the phantom solution, with the material being 6 mm thick on all sides. The full-wave and measurement SNR data included the lossy polycarbonate material, while the MQS simulations used the phantom solution only. Even without the lossy polycarbonate material, there was still a 6 mm distance between the phantom solution and coil elements.

There were some common assumptions for each simulation setup. Two constants, one for all the MQS SNR data and one for all the full-wave SNR data, were used to minimize the RMSE with the measured data as shown in equation (1a), where *c*, S, *M*, and N are the scaling constant, simulated SNR data, measured SNR data, and total number of SNR data samples, respectively. The two scaling constants were found using equation (1b). This scaling approach was used to compensate for any assumption that would result in an SNR mismatch between the simulated and measured SNR data. Regardless of the assumptions, the most accurate simulation method will still have the lowest RMSE. Mismatches between simulated and measured SNR data are expected due to not including the preamplifier or MRI receive chain noise in the total noise calculations, using a uniform 90° excitation (rather than a realistic, nonuniform excitation), and not accounting for signal loss during reception from free induction decay.

(1a) $$\text{RMSE=}\sqrt{\frac{1}{N}\overset{N}{\underset{j=1}{\sum }}{\left(\frac{cS_{j}}{M_{j}}-1\right)}^{2},}$$

(1b) $$c=\frac{\sum_{j=1}^{N}\left(S_{j}/M_{j}\right)}{\sum_{i=1}^{N}{\left(S_{i}/M_{i}\right)}^{2}}.$$

### 2.2. Quasistatic Setup.

MQS simulations were written in Matlab (The Math Works Inc.), and each coil element’s signal and noise were calculated in under 4 seconds. The magnetic and electric fields were calculated using equations (2) and (3), respectively, with *μ*_*o*_, *I*, *dl*, r, and *r*′, being the permeability of free space, the current in the coil element, a vector tangent to the conductor centerline, a vector from the origin to the point where the magnetic or electric field is being calculated, and a vector from the origin to the conductor centerline, respectively [8, 9, 24]. The signal voltage (*V*_*S*_) was calculated using equations (4)–(6) [3, 7]. $B_{1}^{-}$ was calculated using equation (4) with *B*_1*x*_ and *B*_1*y*_ being the *x* and *y* components of the magnetic field. The magnetization was calculated using equation (5) with *M*_*o*_, *ρ*, *γ*, ħ*, B*_*o*_*, K*_*B*_*, T, M*^+^*, α, Tr, T*1, and *T*2 being the magnetization at equilibrium, proton density, gyromagnetic ratio, Planck’s constant divided by 2*π*, static magnetic field strength, Boltzmann’s constant, temperature (Kelvin), magnetization (positive rotating frame), flip angle, repetition time, longitudinal relaxation time, and transverse relaxation time, respectively [7]. No transmit coil simulations were performed. Normally, a transmit coil simulation would be used in determining flip angles, but constant 90° flip angles were assumed instead [25, 26]. The signal voltage was calculated using equation (6) with *ω* being the angular frequency [3].

(2) $$B_{1}=\frac{\mu_{o}I}{4\pi }\oint \frac{\text{d}l\left(r-r^{\prime }\right)}{{\left|r-r^{\prime }\right|}^{3}},$$

(3) $$E=-\frac{\mu_{o}wI}{4\pi }\oint \frac{\text{d}l}{\left|r-r^{\prime }\right|},$$

(4) $$B_{1}^{-}=\frac{{\left(B_{1x}-jB_{1y}\right)}^{*}}{2},$$

(5a) $$M_{o}=\frac{p\gamma^{2}\hbar^{\text{2}}B_{o}}{4K_{B}T},$$

(5b) $$M^{+}=M_{o}\frac{\left(1-e^{-Tr/T_{1}}\right)}{1-\text{cos}\;\alpha \;e^{-Tr/T1}}\text{sin}\;\alpha \;e^{-t/T2^{*}},$$

(6) $$V_{s}=\int_{\text{Voxel}}wM^{+}{\left(B_{1}^{-}\right)}^{*}\text{d}v.$$

The noise voltage (*V*_*N*_) was calculated using equations (7)–(10) [1, 27]. The sample noise (*R*_S_) was calculated using equation (7) with *σ* being the sample conductivity and the electric field per unit current. The coil noise (*R*_*C*_) was calculated using equation (8) with *l, $\hat{\rho }$, D, ω, δ*, and *μ*_*o*_ being the coil element circumference, resistivity, wire diameter, angular frequency, skin depth, and magnetic permeability. The total noise (*R*_*T*_) was calculated using equation (16), and the noise voltage was calculated using equation (10), where *R*_*T*_ and Δ*f* are the noise covariance and bandwidth. The SNR was calculated using equation (11) [1, 8, 28].

(7) $$R_{s}=\sigma \underset{\text{Sample}}{\int }{\left|E\right|}^{2}dv,$$

(8a) $$R_{C}=\frac{l\hat{\rho }}{\pi \left(D-\delta \right)\delta },$$

(8b) $$\delta =\sqrt{\frac{2\hat{\rho }}{w\mu_{o}}},$$

(9) $$R_{T}=R_{S}+R_{C},$$

(10) $$V_{n}=\sqrt{4K_{B}TR_{T}\Delta f},$$

(11) $$\text{SNR}=\frac{V_{s}}{V_{n}}.$$

### 2.3. Full-Wave Setup.

Full-wave simulations were performed using Computer Simulation Technology Microwave Studio (CST MWS) FIT. The simulations had a mesh cell count range of 2–11 million mesh cells, a simulation accuracy of −80 dB, and “Enhanced (Preview)” AR-filter steady state max error of 0.001. CST Design Studio (DS) cosimulation was used to tune and match each coil element.

SNR was determined using signal and noise voltage calculations. The signal voltage was calculated using CST MWS from the magnetic field of each coil element, as generated by one ampere of current in the coil element [1]. B1^−^ field values were calculated using equation (4) while no transmit coil simulations were performed. Constant 90° flip angles were assumed instead. Lastly, equations (5) and (6) were used to calculate the signal [3]. The noise voltage was calculated using equation (10). The total noise was extracted from CST-DS by removing the match capacitors and then measuring the real part of the *z*-parameter [28]. The SNR was calculated for each coil element using equation (11).

Full-wave total noise calculations were performed the same way as was performed on the benchtop. They were measured by removing the match capacitor and measuring the real part of the *Z*_1,1_ parameter. In order for the benchtop and calculated resistances to be equivalent, the active decoupling diode on the match circuit between the signal and ground was included in the CST MWS simulations as a 0.8 pF capacitor.

### 2.4. Experimental Measurement Setup.

SNR measurements were acquired using a MAGNETOM 3T Trio (Siemens Healthcare, Erlangen, DE) MRI scanner [29, 30]. The measurements were made with 2D gradient echo (GRE) axial images with a TE/TR 4.0/500 ms, flip angle 90°, matrix size 320 × 320, FOV 300 × 300 mm, and bandwidth 260 hertz/pixel and noise-only images using the same sequence with the RF transmit voltage set to 0 volts and TR 50 ms. The SNR plots are composed of measurements taken axially through the center of the coil elements. Only one Siemens preamplifier was used for all 24 coil elements. The preamplifier was connected directly into a socket located near the match circuit of the coil element, thereby eliminating any need for a cable between the preamplifier and coil element. The phantom solutions were a mixture of 1.955 g CuSO_4_ × H_2_0 combined with 1.094, 3.020, and 4.915 g NaCl to achieve a permittivity of 78 and conductivity of 0.3, 0.6, and 0.9 S/m at 20°C, respectively. The permittivity and conductivity were measured with an Agilent 85070E dielectric probe kit and Sper Scientific 850037 conductivity pen, respectively. The mass measurements were performed using a Smart Weigh digital jewelry scale GEM20 that is accurate to 0.001 grams. The benchtop total noise measurements were made by removing the match capacitor and measuring the real part of the *Z*_1,1_ parameter [28].

### 2.5. Varied Phantom Sizes Setup.

A simulation experiment was run with four phantoms of varying sizes to simulate the quasistatic error as a function of system size. This setup matched the previous setups, except that there was only one 6 cm coil element and four phantoms of varying sizes, but all with the same conductivity of 0.6 S/m. The phantoms have the following diameter/height in centimeters for the full- (27.8/10.1), half- (13.9/5.1), quarter- (7.0/2.5), and eighth- (3.5/1.3) sized phantoms. There remained a 6 mm gap between the phantom solution and the coil element. Again, the quasistatic simulation was not simulated with the polycarbonate former as was carried out with the full-wave simulations. Lastly, the simulated data for this section were not scaled since they were not being compared to measured data as was carried out in the previous sections.

## 3. Results

**Figure 2:**
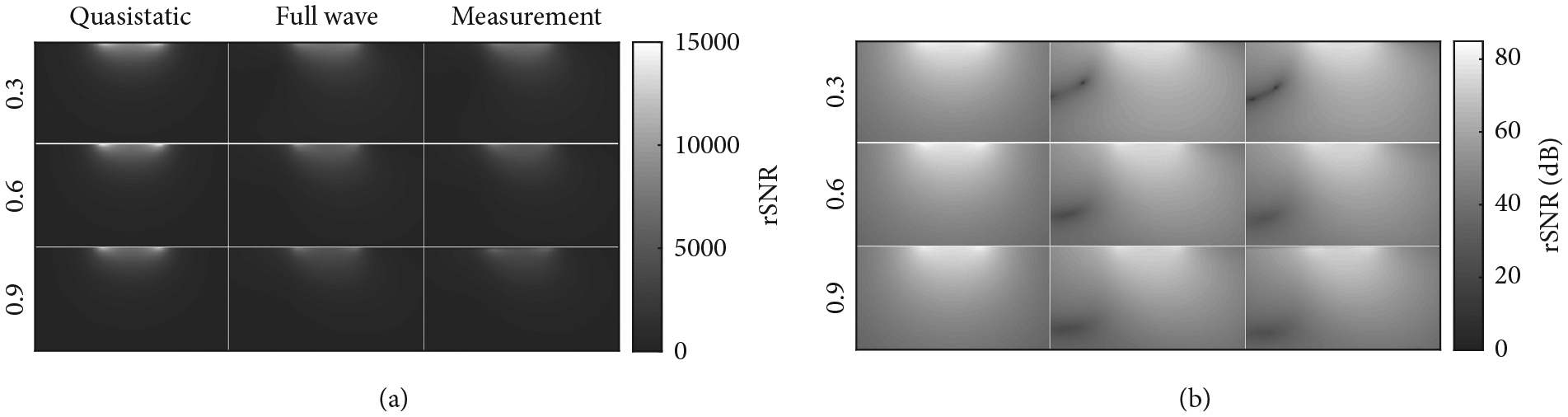
Relative SNR (rSNR) images of quasistatic, full-wave, and measurement SNR data for the 6 cm coil elements. The bottom plots are the logarithmic transformation (dB) of the SNR images. Each image is cropped, so the full phantom is not shown. The dB plots easily show destructive wave effects in the full-wave and measurement images that are not present in the quasistatic images. All coils and phantoms showed these destructive wave effects in full-wave simulations and corresponding measurements. Images were acquired using a 2D axial GRE sequence positioned through the axis of each coil (TE/TR 4.02/500 ms, flip angle 90°, matrix size 192 ×192, FOV 200 × 200 mm, bandwidth 260 Hz/pixel, and slice thickness 5 cm).

**Figure 3:**
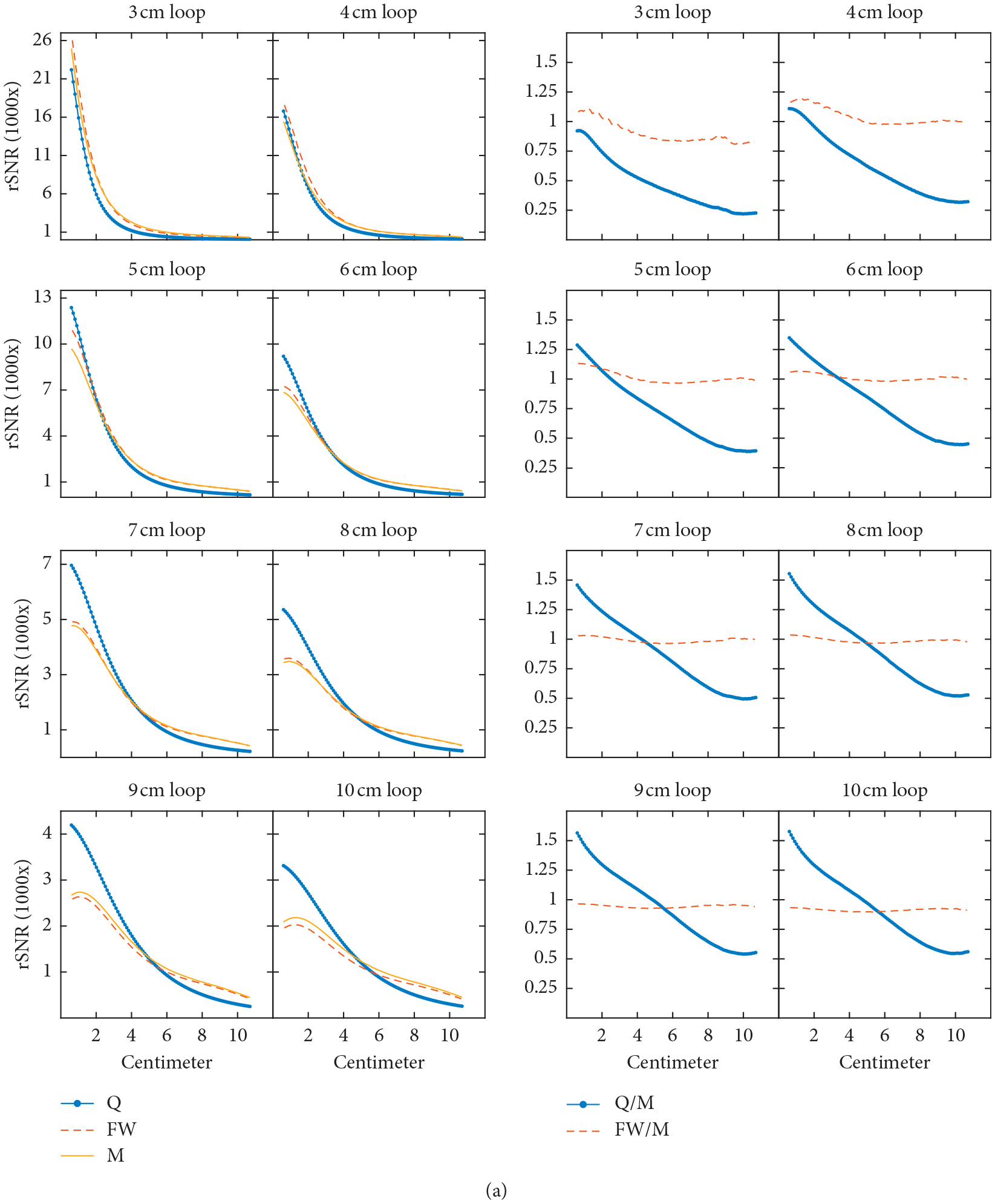

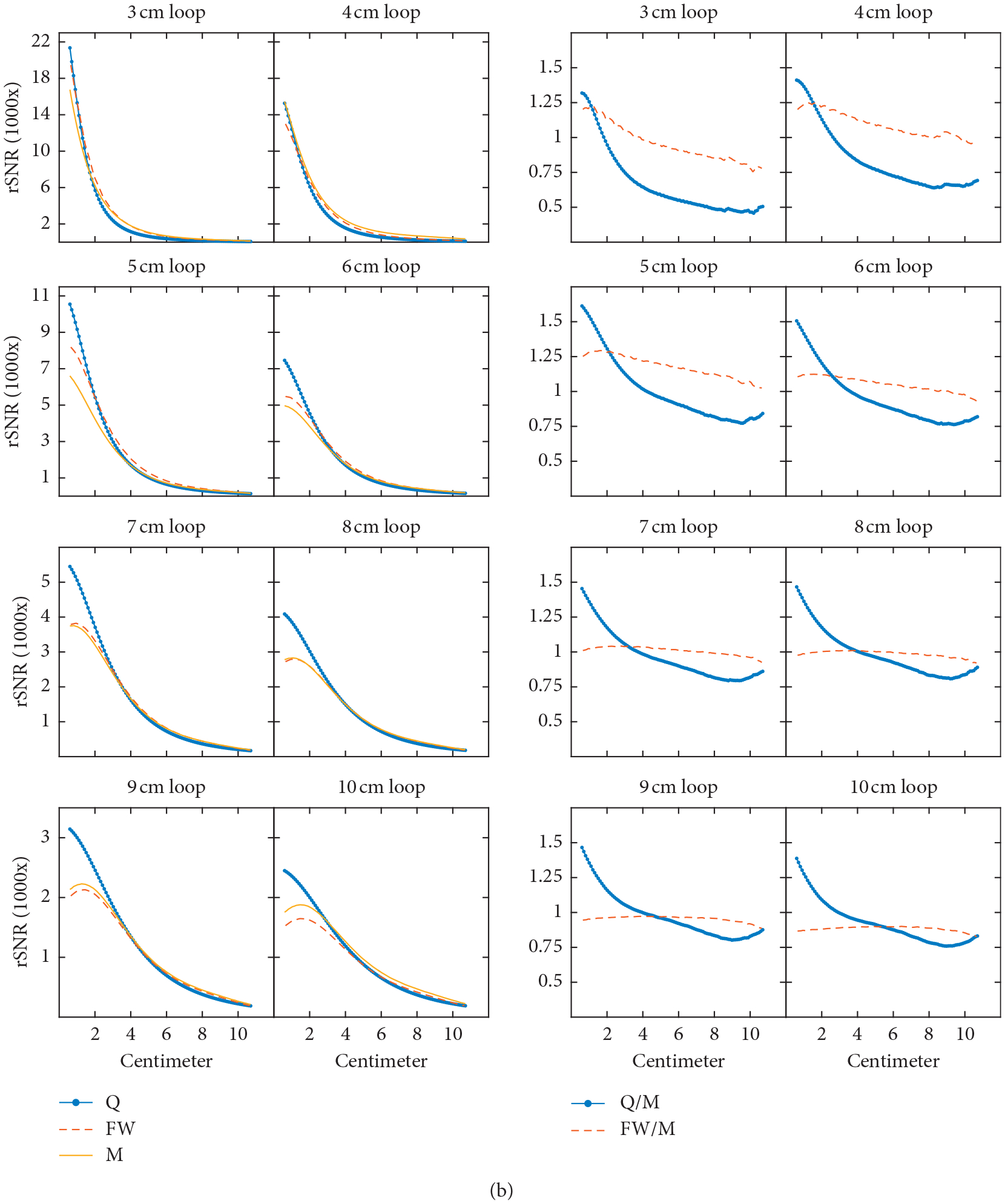

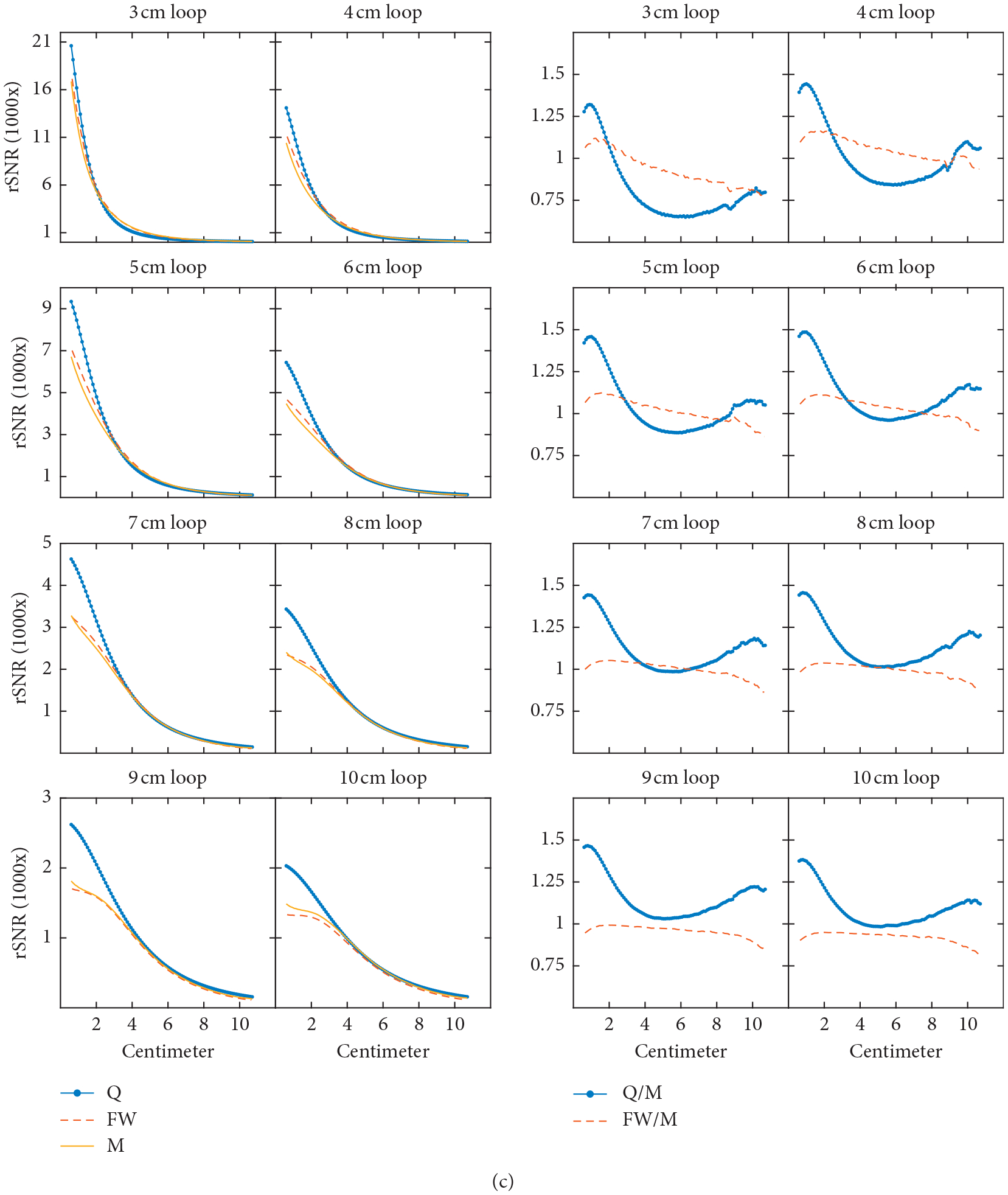
(a) 1D relative SNR (rSNR) plots (left) and 1D rSNR ratio plots (right) of quasistatic (Q), full-wave (FW), and measurement (M) data collected on a 0.3 S/m phantom with data points taken along each coil element’s axis. The coils were separated from the solution by a 6 mm thick lossy polycarbonate former, except that no former was used in the quasistatic simulations. Each legend corresponds to its respective column. (b) 1D relative SNR plots (left) and 1D rSNR ratio plots (right) of quasistatic (Q), full-wave (FW), and measurement (M) data collected on a 0.6 S/m phantom with data points taken along each coil element’s axis. The coils were separated from the solution by a 6 mm thick lossy polycarbonate former, except that no former was used in the quasistatic simulations. Each legend corresponds to its respective column. (c) 1D relative SNR plots (left) and 1D rSNR ratio plots (right) of quasistatic (Q), full-wave (FW), and measurement (M) data collected on a 0.9 S/m phantom with data points taken along each coil element’s axis. The coils were separated from the solution by a 6 mm thick lossy polycarbonate former, except that no former was used in the quasistatic simulations. Each legend corresponds to its respective column.

The RMSE was 0.285 and 0.087 for the MQS and full-wave simulations, respectively, when compared with measured data. Axial SNR plots for the 6 cm coil elements on the 0.6 S/m phantom are shown in Figure 2. Figure 2 shows that the full-wave simulations are able to capture the electromagnetic wave effects that occur when energy is reflected from boundary interfaces while no wave effects are present in the MQS simulations. Simulated SNR line plots and their corresponding SNR ratio plots comparing the simulated to measured SNR data are shown in Figure 3. All SNR line plots were from SNR data sampled along the axis of the coil elements. The maximum and minimum quotient values of the SNR ratio plots were 1.69 and 0.20 for the MQS simulations and 1.29 and 0.75 for the full-wave simulations, respectively.

Total noise (*R*_*t*_) values calculated by MQS and full-wave simulations were compared against benchtop measured noise values; the results are shown in Table 1. The ratio ranges of the MQS and full-wave total noise values compared to the bench measured noise values were 0.27–3.02 and 0.83–1.06, respectively. The full-wave and measurement noise values were recorded with an active decoupling diode (modeled as a 0.8 pF capacitor) connected between the signal and ground on the match circuit, which increased the total noise by approximately 15–30% compared to when the diode was removed.

### 3.1. Varied Phantom Sizes.

**Figure 4:**
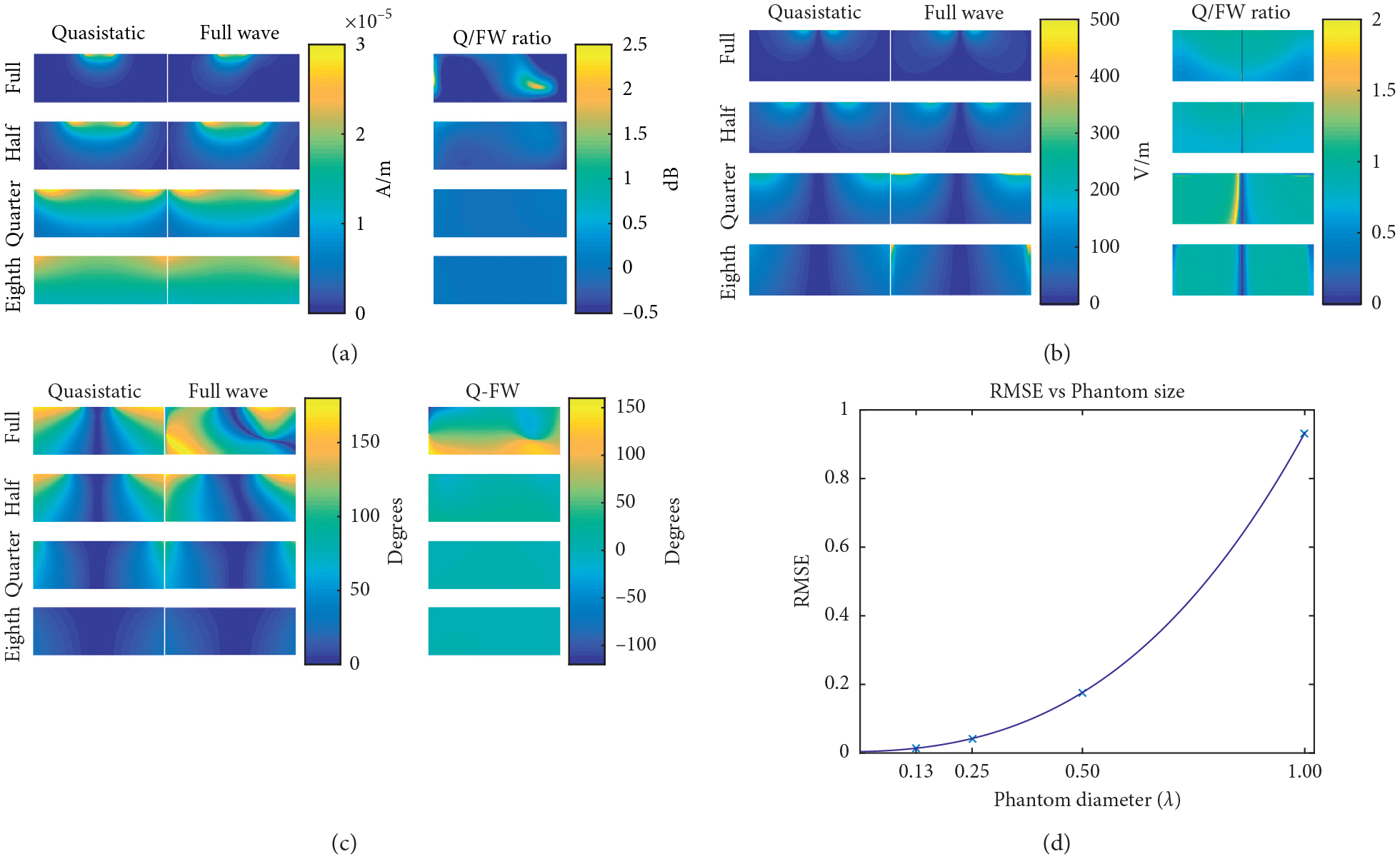
(a) Axial images of the magnetic field magnitude for the quasistatic and full-wave simulations when simulated with a 6 cm diameter coil element on four cylindrical phantoms of varying sizes. The phantoms have the following diameter/height in centimeters for the full (27.8/10.1), half (13.9/5.1), quarter (7.0/2.5), and eighth (3.5/1.3). (b) Axial images of the electric field magnitude for the quasistatic and full-wave simulations when simulated with a 6 cm diameter coil element on four cylindrical phantoms of varying sizes. (c) Axial images of the phase for the quasistatic and full-wave simulations when simulated with a 6 cm diameter coil element on four cylindrical phantoms of varying sizes. (d) Plot showing the convergence of the quasistatic method to the full-wave method as sample size is reduced. The RMSE was calculated using equation (1a)) with *c* equal to one and S and *M* being the magnitude of the magnetic fields of the quasistatic and full-wave simulations, respectively. The data points were fit with a third-order polynomial, starting with the leading coefficient; the coefficients are 0.51, 0.38, 0.02, and 0.00. For reference, see Figure 4(a).

Magnetic, electric, phase, and RMSE results as the phantom size was decreased are shown in Figure 4. As can be seen, the differences between the quasistatic and full-wave simulations are diminished as the size of the phantom is decreased.

## 4. Discussion

This paper compared MQS and full-wave simulated SNR data to measured SNR data to determine how accurately the two simulation methods predict measured SNR data. This was carried out with varying circular coil sizes so that each coil element would have varying ratios of coil-to-sample noise. This, along with phantoms that absorbed RF power at differing rates, provided the variability necessary to demonstrate whether the simulations would be capable of predicting the complex wave behavior of each test case and, in turn, predict the correct signal and noise values.

The assumption that “the system is small compared to the electromagnetic wavelength associated with the dominant time scale” was not met for the MQS model applied to the large phantom because the phantom diameter was on the order of a wavelength for the received radio-frequency signal. The Darwin model may be used to yield slightly more accurate results. However, the Darwin model would not resolve any inaccuracies caused by not accounting for the wave effects.

The MQS SNR data did not match the measured SNR data as closely as that of the full wave. The full-wave RMSE was a factor of 3 smaller than that of the MQS. This was in large part due to the MQS simulations not accounting for standing waves, while the full-wave simulations did, as seen in Figure 2. The full-wave simulations account for reflections at the interface of the solution, phantom, and air while the MQS simulations assume no boundary conditions. Therefore, the MQS method has no reflections and, therefore, no standing waves. The lack of constructive and destructive interference accounts for the MQS method over- and underpredicting received signal values.

The MQS simulations did not accurately predict total noise values, while the full-wave simulations did, when the coil element noise was dominated by sample noise. As the coil elements increase in size, the reflections and standing waves cause the total noise value of the 10 cm coil elements on the 0.3 S/m phantom to exceed that of the 0.6 and 0.9 S/m. This phenomenon is captured by the full-wave simulations, but not the MQS simulations. The total noise results in Table 1 show that as coil element diameter increases and the noise becomes sample noise dominated, the full-wave simulations can more accurately predict the total noise. The FIT method used does not fully account for current density distribution variations as a function of conductor thickness or skin effect when determining coil noise [13, 31, 32]. If only sample noise dominated coil elements were used in the full-wave simulations, then the RMSE value most likely would have been lower than 8.7%. More work would need to be conducted to determine how accurate the simulations could be in this case. Also, the 6 mm thick lossy polycarbonate former and the active decoupling diode (modeled as a 0.8 pF capacitor) were required to match the full-wave noise values to the benchtop noise values.

Shrinking the size of the sample so it is small compared to the RF wavelength reduced the difference between the MQS method and the full-wave method. The magnetic field and phase difference became nearly negligible when the eighth phantom was used. The difference between the electric fields of the two simulation methods, on the smaller phantoms, is most likely due to the lack of a polycarbonate former when simulating the MQS method. The phase is important for applications such as parallel imaging, but more specifically calculating geometry factor (g-factor) maps. The wave effects, such as those seen in the full-sized phantom, would need to be considered if the g-factor map was to be calculated accurately. Otherwise, a smaller phantom could be used to generate accurate results. This study was limited by a one-sized phantom with 8 coil elements of differing sizes, but future work could involve finding the MQS RMSE value for various sample sizes and comparing them to that of the full-wave simulations. If smaller sample sizes yield MQS RMSE values comparable to those of the full wave, then MQS simulations could potentially be used instead.

## 5. Conclusions

This work compared simulated SNR data, determined from magnetic and electric field calculations using magnetoquasistatic and full-wave simulations. The simulated to measured SNR data were compared to determine the RMSE, and the magnetoquasistatic RMSE was approximately 3x larger than the full-wave RMSE. The requirement that “the system is small compared to the electromagnetic wavelength associated with the dominant time scale” was not met by the magnetoquasistatic simulations of all 8 coils on the full-size phantom for all conductivities, meaning only the full-wave simulations were able to account for wave effects in the sample and, therefore, able to characterize the sample noise. The accuracy of the MQS method improved as the relative phantom size decreased. Skin effect and the associated coil noise, however, were not fully assessed with the full-wave simulations used in this study.

## Figures and Tables

**Table 1:** Total noise (Ohms).

Circular loop diameter	3 cm	4 cm	5 cm	6 cm	7 cm	8 cm	9 cm	10 cm
*Quasistatic (Q) calculations*								
0.3 S/m	1.51	1.77	2.21	2.88	3.82	5.05	6.63	8.56
0.6 S/m	1.65	2.16	3.02	4.33	6.18	8.63	11.8	15.6
0.9 S/m	1.79	2.54	3.82	5.78	8.54	12.2	16.9	22.6
*Q/B ratios*								
0.3 S/m	3.02	1.36	0.84	0.61	0.46	0.39	0.31	0.27
0.6 S/m	2.58	1.58	1.09	0.89	0.78	0.73	0.67	0.65
0.9 S/m	2.49	1.59	1.17	1.07	0.99	0.95	0.89	0.92
*Full-wave (FW) calculations*								
0.3 S/m	0.48	1.13	2.39	4.52	7.99	13.2	20.9	31.9
0.6 S/m	0.53	1.21	2.49	4.48	7.52	11.8	17.8	25.6
0.9 S/m	0.62	1.41	2.86	5.04	8.26	12.6	18.5	25.8
*FW/B ratios*								
0.3 S/m	0.96	0.87	0.91	0.97	0.97	1.01	0.99	1.00
0.6 S/m	0.83	0.88	0.9	0.92	0.95	0.99	1.01	1.07
0.9 S/m	0.86	0.88	0.87	0.93	0.96	0.98	0.97	1.04
*Benchtop (B) measurements*								
0.3 S/m	0.50	1.30	2.63	4.67	8.26	13.1	21.2	31.9
0.6 S/m	0.64	1.37	2.76	4.86	7.93	11.9	17.7	24.0
0.9 S/m	0.72	1.60	3.27	5.42	8.60	12.8	19.0	24.7

## Data Availability

Data are available on request by contacting either J. Rock Hadley at rock.hadley@utah.edu or Michael *J* Beck at mjb27@utah.edu.
